# Immune Checkpoint Inhibitor-Associated Celiac Disease: A Retrospective Analysis and Literature Review

**DOI:** 10.3390/diseases12120315

**Published:** 2024-12-03

**Authors:** Malvika Gupta, Christopher Graham, Supriya Gupta

**Affiliations:** 1 Kasturba Medical College, Manipal Academy of Higher Education, Mangalore 575001, India; malvikaguptamd@gmail.com; 2 Division of Hematology, Oncology and Transplantation, University of Minnesota, Minneapolis, MN 55455, USA; grah0329@umn.edu

**Keywords:** immunotherapy, celiac disease, enterocolitis, CTLA-4, HLA-DQ2, PD-L1, PD-1, adverse effects, gluten-free diet

## Abstract

Introduction: Immune checkpoint inhibitors (ICI) are used to treat various malignancies. They block the inhibitory signals of tumor cells and enhance the inflammatory cascade, which results in tumor killing. However, this can lead to unchecked inflammation throughout the body, leading to various adverse effects. A rare gastrointestinal adverse effect of ICI therapy is the development of immune-mediated celiac disease. This entity has a similar clinical presentation to the more common ICI-induced enterocolitis. Our study aims to determine the clinical characteristics and optimal treatment strategies for this rare ICI toxicity and differentiate it from ICI-induced enterocolitis. Methods and Material: We conducted a retrospective analysis of eight cases of ICI-induced celiac disease and 24 cases of ICI-induced enterocolitis from the literature. Data on patient demographics, clinical history, therapeutic interventions and outcomes were collected. A comparative analysis was performed to identify the key differences between the two groups. Results: Patients with ICI-induced celiac disease were more likely to have a pre-existing autoimmune condition and HLA-DQ2 positivity. Significant differences in clinical manifestations, histological findings, and treatment outcomes were observed. Notably, weight loss, nutritional deficiencies and electrolyte abnormalities were more commonly associated with ICI-induced celiac disease. Regarding pathology, duodenal villous blunting was noted more commonly with ICI-induced celiac disease. Initiating a gluten-free diet led to a rapid improvement in patients with ICI-induced celiac disease, while immunosuppressive therapy did not have an impact. Conclusion: ICI-induced celiac disease is a rare and underrecognized gastrointestinal adverse effect of ICI therapy, often misdiagnosed as ICI-induced enterocolitis. Early recognition and treatment with a gluten-free diet can lead to rapid symptom resolution, sparing patients from unnecessary systemic immunosuppression and the discontinuation of antineoplastic immunotherapy.

## 1. Introduction

The immune regulatory proteins, cytotoxic T lymphocyte antigen (CTLA-4), programmed cell death protein (PD-1) and its ligand, PD-L1, are important immune system regulators known as immune checkpoint receptors and are pivotal in the pathogenesis of cancer development [1]. Humanized monoclonal antibodies have been developed, which target immune checkpoint receptors, including monoclonal antibodies blocking CTLA-4 (Ipilimumab), PD-1 (Pembrolizumab, Nivolumab) and PDL-1 (Atezolizumab, Avelumab and Durvalumab), allowing for immune-driven cancer killing [2]. Immune checkpoint inhibitors (ICIs) have been approved by the Food and Drug Administration (FDA) for the treatment of various malignancies, including melanoma, non-small cell lung carcinoma, squamous cell carcinoma of the head and neck, renal cell carcinoma, colorectal carcinoma, classic Hodgkin’s lymphoma, Merkel cell carcinoma, urothelial carcinoma, etc. [2,3].

ICIs bind to and prevent the interaction of PD-L1 on tumor cells with PD-1 on cytotoxic T-cells. This leads to increased cytotoxic T-cell activity, the production of cytokines and the enhancement of the inflammatory cascade, targeting tumor cells [4]. While this may be desirable against neoplastic cells, it may lead to unregulated inflammation throughout the body, leading to various adverse effects [4]. This autoimmune-like toxicity can involve multiple organ systems, including the skin, gastrointestinal tract and endocrine glands [5]. Known gastrointestinal adverse effects include enterocolitis, hepatotoxicity and pancreatitis, which are well described [3]. In one study looking at ICI adverse events, it was found that with Ipilimumab, gastrointestinal adverse effects occurred in 39.7% of the cases. Of these patients, 73.11% presented with colitis/enterocolitis, 18.27% presented with hepatitis and 2.15% presented with pancreatitis. In contrast, none of the patients who received pembrolizumab or nivolumab had gastrointestinal adverse effects [6].

Celiac disease is a common autoimmune disease of the small intestine. The global incidence of celiac disease based on seroprevalence is 1.4%, with a biopsy-confirmed prevalence of 0.7% [7]. It affects 1 in 200 individuals and is found more commonly in Western societies. [8]. It is caused by sensitivity to dietary gluten in genetically at-risk individuals [9]. The strongest genetic susceptibility for celiac disease is seen in individuals who carry the class-II human leukocyte antigen (HLA) genes-HLA-DQ2 and DQ8 [10]. Classic clinical manifestations include bulky, floating, foul-smelling, loose stools [9]. Extra-intestinal manifestations of celiac disease include dermatitis herpetiformis and atrophic glossitis [9]. It is primarily diagnosed by serological testing for anti-tissue transglutaminase IgA (sensitivity of 93% and specificity of ≥98%), anti-endomysial antibody IgA (sensitivity of 93% and specificity of >99%) and anti-deamidated gliadin peptide IgA and IgG (sensitivity of 75% and specificity of 94%) and confirmed by duodenal biopsy [2,3]. Histological features of celiac disease include intraepithelial lymphocytosis (>25 T-lymphocytes which are CD3-positive per 100 enterocytes), crypt hyperplasia and villous atrophy (a villous/crypt ratio of less than 3:1) in the duodenal mucosa [2]. The standard treatment for celiac disease is a gluten-free diet, which resolves clinical symptoms and returns normal duodenal microscopy [7]. The overall mortality from celiac disease is estimated to be higher than that of the general population, with a hazard ratio of 2.0 (95% CI, 1.5–2.7) and is seen more commonly in patients with malabsorption symptoms [11]. Biagi et al. suggested that the mortality rate in patients with celiac disease correlated with the amount of gluten consumed before and after diagnosis along with the extent of the lesion in the small intestine, with increased mortality seen in patients who developed complications of celiac disease such as gastrointestinal malignancies [12].

ICI-induced celiac disease is a rare gastrointestinal adverse effect of immune checkpoint inhibitor therapy. One study found that the incidence of ICI-induced celiac disease in patients with melanoma treated with ICIs was only 0.3% [13]. To date, only eight cases of ICI-induced celiac disease have been reported in the literature. The pathophysiology, risk factors, clinicopathological presentation and management are not well understood. Our study aims to determine the clinical characteristics and risk factors for developing ICI-induced celiac disease. Additionally, due to the common clinical presentation between ICI-induced celiac disease and the more common ICI-induced enterocolitis, our study aims to deduce the difference between the two ICI-associated gastrointestinal toxicities and determine the optimal treatment strategies.

## 2. Material and Methods

We conducted a retrospective observational study that evaluated the epidemiological features, clinicopathological profile, treatments and outcomes of patients with ICI-induced celiac disease. Database searches through the National Library of Medicine (NLM) were carried out using the following searches: “immune checkpoint inhibitor-associated celiac disease”, “celiac disease with immune checkpoint inhibitors”, “case reports on celiac disease with immune checkpoint inhibitors”, “celiac disease with pembrolizumab”, “celiac disease with nivolumab”, “celiac disease with atezolizumab”, “celiac disease with Durvalumab”, “celiac disease with Avelumab” and “celiac disease with ipilimumab”.

We compiled a database of all eight cases of ICI-induced celiac disease published in the literature to perform this analysis [3,4,13,14,15,16,17,18]. The diagnostic criteria used for the selection of the cases included patients undergoing treatment for malignancy with immune checkpoint inhibitors, who presented with non-bloody diarrhea after starting treatment with ICIs and who had duodenal biopsies suggestive of celiac histology supported by positive celiac serology. Initial upper gastrointestinal endoscopies and biopsies were performed to rule out ICI-induced enterocolitis and evaluate for alternative diagnoses due to the lack of a response to the conventional management of diarrhea (3/8 with supportive care and 5/8 with systemic steroids) and unremarkable colonoscopy exams. Further evaluation with upper gastrointestinal endoscopies revealed celiac histology. This diagnosis was supported further by positive celiac serology in all eight patients.

We also compiled data from 24 published cases of ICI-induced enterocolitis for comparison [19,20,21,22,23,24,25,26,27,28,29,30,31,32,33,34,35,36,37,38,39,40,41,42]. The diagnostic criteria used for the selection of the cases included patients undergoing treatment for malignancy with immune checkpoint inhibitors, patients who presented with non-bloody diarrhea after starting treatment with ICIs and patients who had a colonoscopy suggestive of colitis and a negative celiac serology. Data on patient demographics, clinical history, medication history, presenting symptoms, relevant past medical history, family history, treatment and outcome were collected in a pooled database.

Descriptive statistics were used to evaluate patient and disease characteristics, therapeutic interventions and outcomes between the cases (ICI-induced celiac disease) and controls (ICI-induced enterocolitis). Data collected included demographic characteristics, the primary malignancy, the disease stage, the treatment before initiating ICI, a preexisting autoimmune condition, the type of ICI used, the number of cycles before the development of toxicity, the time from the first dose to the development of toxicity, clinical features, microscopic features, genetic testing for HLA DQ2, therapeutic interventions and outcomes (response to therapy, resolution of symptoms, time to improvement and re-initiation of ICI therapy).

A chi-square test was used to determine if there is an association between specific risk factors, clinical and pathological manifestations and outcomes while comparing the two groups. A *p*-value of ≤0.05 was considered significant. We also calculated the odds ratio (OR) with a 95% confidence interval (95% CI) for potential risk factors of ICI-induced celiac disease. A confidence interval excluding 1.000 and a *p*-value of ≤0.05 was considered significant in determining prognostic factors in ICI-induced celiac disease.

## 3. Results

Eight cases of ICI-induced celiac disease (ICI-CD) and twenty-four cases of ICI-induced enterocolitis (ICI-EC) were included.

### 3.1. Demographic Characteristics

The median ages for the ICI-CD cohort and ICI-EC cohort were 70 years (62–79 years) and 65 years (22–83 years), respectively. A higher proportion of males were observed in the ICI-EC cohort (75% or N = 18) compared to the ICI-CD cohort (62.5% or N = 5) (*p* = 0.496). Of the patients with ICI-CD, 50% (N = 4) were Caucasian, compared to 20.83% (N = 5) of patients with ICI-EC (*p* = 0.389). One patient (4.17%) in the ICI-EC cohort was Asian (Table 1).

Patients with ICI-CD had a 7.67 times higher rate of preexisting autoimmune disease compared to the ICI-induced enterocolitis cohort (25% vs. 4.17%, *p* = 0.08). Only three patients (37%) in the ICI-induced cohort were tested for HLA-DQ2, with all three testing positive. None of the ICI-induced enterocolitis patients were tested for HLA-DQ2.

The most common primary malignancy in the ICI-CD cohort was melanoma (37.5%), followed by genitourinary cancers (25%), non-small cell lung cancers (NSCLC) (12.5%) and others (25%). For patients who had ICI-EC, the most common malignancies were melanoma (50%), NSCLC (20.83%), genitourinary cancers (16.7%), gastrointestinal cancers (8.3%) and other malignancies (4.17%) (Table 1). The most common stage for patients with cancer was Stage IV for both the ICI-CD and ICI-EC groups (75% vs. 50%). The distribution between Stages I and III was similar between the ICI-CD and ICI-EC groups. There was no statistically significant difference between the two groups based on primary malignancy and stage of malignancy.

### 3.2. Pathology

On duodenal microscopy, 100% of patients with ICI-CD had villous blunting compared to 16.67% (N = 4) of patients with ICI-EC (*p* = 0.078). Crypt hyperplasia (37% vs. 8%), lamina propria expansion (25% vs. 4.17%) and CD3 positivity (25% vs. 4%) were more commonly seen with ICI-CD (Table 1). On colonic microscopy, no patients with ICI-CD had cryptitis, compared to 54% of ICI-EC patients (*p* = 0.004). Inflammatory infiltrates in the lamina propria (54% vs. 0%, *p* = 0.0036) and apoptotic bodies (21% vs. 12.5%) were seen more frequently in the ICI-EC cohort.

### 3.3. Prior Therapy

There was no difference in incidence among patients who received surgical resection before ICI between the two groups (37.5% for both). More patients in the ICI-CD cohort had chemotherapy before ICI compared to the ICI-EC cohort (37.5% vs. 29%, *p* = 0.660) and those in radiation therapy (37% vs. 16%, *p* = 0.21). Only 12.5% in the ICI-CD cohort and 21% in the ICI-EC cohort had prior ICI. The use of single-agent Ipilumumab, Pembrolizumab and Nivolumab and combination Ipilumumab + Nivolumab prior to developing ICI toxicity was similar between the groups, with a median of two prior cycles for each before the onset of toxicity. One patient with ICI-CD had received durvalumab compared to none for ICI-EC. There were no reported cases of ICI-CD with Atezolizumab or Avelumab.

### 3.4. Immunotherapy-Related Adverse Event (irAE)

For patients with ICI-CD, the timing of diarrhea occurred earlier at 3 weeks compared to 6 weeks for IEC-EC. The occurrence of diarrhea in the ICI-CD cohort did not lead to ICI treatment interruptions or discontinuation compared to patients with ICI-EC (50% vs. 100%, *p* = 0.02).

Grade IV Diarrhea, as graded by Common Terminology Criteria for Adverse Events Version 5.0 (CTCAE v5), was higher in patients with ICI-CD compared to ICI-EC (37% vs. 20.8%, *p* = 0.5). For non-diarrhea-associated irAEs, abdominal pain was five times more likely to occur with ICI-EC compared to ICI-CD (62.5% vs. 12.5%, *p* = 0.014). Weight loss was three times (95% CI; 1.16–7.73) more likely to occur with ICI-CD compared to ICI-EC (62.1% vs. 25%, *p* = 0.027). Nutritional deficiencies and electrolyte abnormalities also occurred 2.6 times more frequently (95% CI; 1.2–5.4) with ICI-CD compared to ICI-EC (75% vs. 29%, *p* = 0.02).

### 3.5. Treatment

Therapeutic interventions included treatment with a gluten-free diet, topical glucocorticoids in the form of budesonide and systemic glucocorticoids such as prednisone and the use of immunosuppressive therapy including Infliximab, Vedolizumab and Tacrolimus.

Patients with ICI-CD were treated with systemic glucocorticoids as the first-line treatment in 62.5%, with a median dose of 50 milligrams (mg) per day in Prednisone equivalents, compared to 91.3% (N = 21) of patients with ICI-EC at a median dose of 65 mg per day (*p* = 0.116). Three (37%) patients with ICI-CD received a gluten-free diet as the first-line treatment and had rapid clinical improvement. Five patients (60%) were started on corticosteroids as the first-line treatment, though they did not show improvement in their symptoms until starting a gluten-free diet later. Two patients (25%) received steroids concurrently with a gluten-free diet and both improved clinically. No additional treatment was required for the patients in the ICI-CD cohort after initiating a gluten-free diet. None of the ICI-CD patients required biologics for the management of their symptoms.

For patients with ICI-EC, 91% received systemic steroids as a first-line treatment, with 48% having a resolution of symptoms (*p* = 0.261). One patient each received supportive care and topical NSAIDS (mesalamine plus octreotide and 5-Aminosalicylate, respectively) without improvement. Biologics were administered to 39%: eight received Infliximab, of which six received it as a second-line treatment, and 75% responded. Out of the non-responders, Vedolizumab was started as the next line of therapy (one responded and one was refractory, requiring tacrolimus). One patient received vedolizumab instead of Infliximab due to concerns for toxicity with Infliximab and showed improvement (Table 2).

The median time for symptomatic improvement after initiating treatment was 14 (1–21) days for the ICI-CD cohort and 4 (1–42) days for the ICI-EC cohort. Two patients with ICI-CD were reinitiated on ICI therapy after the resolution of their symptoms, with 50% relapsing afterwards despite gluten-free diet adherence. For patients with ICI-EC, three patients (13%) were reinitiated on ICI therapy, and none of them had a relapse.

## 4. Discussion

Pathogenesis of Celiac Disease: The primary pathogenesis of celiac disease is an exaggerated immune response to gluten and similar proteins found in everyday food grains like wheat, rye and barley. This inappropriate immune response leads to the release of interferon-gamma, interleukin-15 and cytokines into the duodenal mucosa [7,8]. These cytokines play a role in mobilizing T-helper cells, which recognize gluten peptides modified by the mucosal enzyme transglutaminase. Therefore, the modification of gluten by tissue transglutaminase is a critical event in the development of celiac disease. Serologically, the presence of autoantibodies in tissue transglutaminase indicates celiac disease [8]. Celiac disease is known to be overrepresented in patients with other autoimmune diseases like type-1 diabetes mellitus and thyroiditis [8]. Type 1 diabetes mellitus, in particular, is associated with the same genetics as celiac disease [9]. Therefore, celiac disease screening is recommended for these patients [8]. Our study shows that patients with preexisting autoimmune disease, specifically thyroiditis or type-1 diabetes mellitus, were more likely to develop ICI-CD.

Role of Genetics in the Pathogenesis of Celiac Disease: A high prevalence among first-degree relatives of patients with celiac disease indicates that genetic factors play a role in susceptibility [43]. Multiple genes have been implicated in the pathogenesis of celiac disease, particularly *HLA-DQ* and *CTLA-4*. The majority of patients with celiac disease carry the HLA-DQA1*05 and HLA-DQB1*02 genes that encode the molecule HLA-DQ2 which binds to the peptide fragments of gluten proteins and presents them to T helper cells, leading to activation, cytokine release and infiltration of the mucosal border: the direct result is villous atrophy and crypt hyperplasia affecting the absorption of micro- and macronutrients [8]. However, there is a difference in the concordance rate of celiac disease between HLA-identical siblings (30%) and monozygotic twins (70%), suggesting that non-HLA genes may be implicated in celiac disease as well [44]. According to one study, untreated patients with celiac disease had high cytotoxic T lymphocyte antigen-4 (CTLA-4) concentrations in their serum [3]. As previously discussed, CTLA-4 is also known to influence T-cell activation and thus is thought to be a non-HLA gene contributor to celiac disease and is the target for ipilimumab [8]. Multiple population studies have concluded that polymorphism in the CTLA-4 exon is a non-HLA determinant in developing susceptibility to celiac disease [45,46,47,48,49]. CTLA-4 and CD28 are essential modulators of T-cell function. Both bind to B7 molecules on antigen-presenting cells. While the binding of CD28 is required for T-cell activation, CTLA-4 maintains tolerance and induces anergy by negative regulation. Variations in CTLA-4 have been implicated in many autoimmune conditions, including celiac disease [46].

Pathogenesis of ICI-induced celiac disease: It is hypothesized that immune cell activation with ICI therapy leads to the unmasking of gluten sensitivity in susceptible individuals and subsequent T cell-mediated tissue injury [15]. Patients who develop ICI-induced celiac disease may have a variant of celiac disease with cross-tolerance and decreased penetrance that is unrestricted by the blockade of CTLA-4 [1]. In one study, 25% of patients with ICI-induced celiac disease had a family history of celiac disease, supporting this hypothesis [1,16]. In our study, three patients with ICI-induced celiac disease underwent HLA-DQ2 testing, and all of them tested positive. Additionally, the presence of concurrent autoimmune disease was more likely in patients who developed ICI-induced celiac disease. The primary site to be affected was the duodenum, sparing the rest of the GI tract, suggesting a separate mechanism than traditional enterocolitis associated with ICI use. However, confirmation with endoscopies and tissue-transglutaminase IgA should be considered in patients with factors that put them at a higher risk for ICI-CD prior to starting ICI therapy [16].

Risk Factors for ICI-CD: In our study, we observed that the median age of patients with ICI-CD and ICI-EC was similar, around 65 to 70 years. Variations in sex chromosomes and hormonal changes make females more at risk for autoimmune conditions [50]. This may explain why women had a higher likelihood of having ICI-induced celiac disease than ICI-induced enterocolitis in our study. Studies have indicated that prolonged treatment with ICIs does not result in an increased incidence of ICI-induced toxicities [14]. This was confirmed in our study, as the median number of cycles of ICI therapy was two, ranging between one and five in the ICI-induced celiac disease cohort. Only 25% of the patients received more than two cycles of ICI therapy before presenting with diarrhea. Most (75%) of the cohort received one or two cycles only. Additionally, patients could present with diarrhea any time between one and fifteen weeks after administering the first dose of ICI.

Clinical presentation of celiac disease: Falade et al. reported a case of a patient treated with combined CTLA-4 and PD-1 inhibition for metastatic melanoma who developed a fulminant manifestation of celiac disease with severe protein-losing enteropathy, resulting in hypotension and anasarca. The patient also presented with transaminitis, which was secondary to celiac disease and not concomitant with ICI-induced hepatotoxicity. The patient improved with supportive management and a gluten-free diet. No systemic immunosuppression was given. The fulminant nature of ICI-induced celiac disease is uncommon but known to occur [13]. Therefore, it is essential to include it in the differential diagnosis for ICI-induced gastrointestinal toxicity, irrespective of the severity of the presentation. Not all patients with celiac disease manifest with diarrhea. Silent clinical features of celiac disease can include iron deficiency anemia and osteoporosis. Patients may present with fatigue, depression and infertility and not point towards gastrointestinal disease at all [14,43]. Therefore, monitoring asymptomatic patients on ICI therapy for nutritional deficiencies is important. In our study, abdominal pain was more likely to manifest in patients with ICI-EC. In contrast, weight loss, nutritional deficiencies and electrolyte disturbances were more likely to be seen in patients with ICI-CD. Extra-intestinal manifestations were not observed in our study in the ICI-CD cohort.

Histology of gastrointestinal adverse effects of ICIs: Colitis is the most frequent manifestation of ICI-induced gastrointestinal adverse effects [2]. Macroscopically, there may be erythema, granularity and mucosal ulcers. Histologically, diffuse active colitis patterns may be seen with CTLA-4 inhibitors and lymphocytic and collagenous colitis patterns may be seen with pembrolizumab [19]. In the duodenum, ICI-related toxicity may manifest as erythema, erosions, ulcers or strictures macroscopically. Histological findings may vary from normal villous architecture to the severe blunting of villi, increased lamina propria inflammation, intraepithelial lymphocytosis and scattered apoptotic bodies [19]. Regarding the immunophenotypic profile, the classical celiac disease shows increased CD3+, CD8+ and *γ*δ T-cell intraepithelial lymphocytosis. On the other hand, ICI-CD has more CD68+ and PD-L+ macrophages in the lamina propria compared to classic celiac disease [15].

A retrospective analysis conducted by Fazal et al. studied 40 patients who presented with diarrhea following treatment with ICIs targeting PD-1. Of these patients, 17.5% had macroscopic evidence of duodenal inflammation and 71% of those patients had microscopic evidence of villous atrophy. However, serological evidence of celiac disease with anti-tTG IgA was not commented upon [4]. Our study showed that colon biopsies were more likely to show evidence of cryptitis, crypt abscesses and inflammatory infiltrates in the lamina propria in ICI-EC than in ICI-CD. On the other hand, both cohorts showed evidence of villous blunting and crypt hyperplasia on duodenal biopsy, although this was more common in ICI-CD.

Comparison between ICI-EC of the duodenum and ICI-CD: There are subtle histological features that may point more towards ICI-EC involving the duodenum rather than ICI-CD. These include patchy intraepithelial lymphocytosis and more neutrophilic or eosinophilic infiltrate in the lamina propria [2]. However, serological testing is crucial to distinguishing between the two entities [50,51]. In a study conducted by Irshaid et al., comparing celiac disease, ICI-EC involving the duodenum demonstrated more neutrophilic infiltrate compared to celiac disease. It also showed increased CD3+ lymphocytes, increased CD8+ lymphocytes and a reduced CD4:CD8 ratio in the lamina propria when compared to ICI-CD [52]. However, there was no significant difference in the degree of inflammation of lamina propria, intraepithelial lymphocytosis, crypt hyperplasia, apoptotic bodies or lymphoid aggregates [52]. Badran et al. conducted a study comparing the clinical–epidemiological features of patients presenting with ICI-EC of the duodenum and ICI-CD. They concluded that the mean anti-tTG IgA levels in patients with ICI-EC duodenitis was 1.3 ± 0.23 units. In contrast, for patients with ICI-CD, the mean level was 121.21 ± 80.29 units (*p* = 0.003). The similarities in the clinical presentations between the two entities suggest that the immunological mechanism drives these two processes. However, the lack of improvement with a gluten-free diet and the negative serology for anti-tTG suggest different antigenic targets between the two entities [1,12].

Other differential diagnosis of ICI-CD: Histological features of celiac disease include intraepithelial lymphocytosis (>25 T-lymphocytes which are CD3-positive per 100 enterocytes), crypt hyperplasia and villous atrophy (a villous/crypt ratio of less than 3:1) in the duodenal mucosa [2]. Differential diagnosis for villous atrophy with intraepithelial lymphocytosis includes infection with norovirus and cryptosporidiosis, tropical sprue, collagenous sprue, drugs such as angiotensinogen-converting enzyme inhibitors (ACEi) and non-steroidal anti-inflammatory agents, IgA deficiency, human immunodeficiency virus-related enteropathy and small intestine bacterial overgrowth [7]. Other drugs can present with chronic diarrhea, which must be kept in mind as a differential diagnosis. Olmesartan, an ACEi, and mycophenolate mofetil increase inflammation and enteropathy and can lead to chronic diarrhea [53]. Many antibiotics can also induce diarrhea by decreasing the digestive function of colonic microbiota. Chemotherapeutic agents may damage the gastrointestinal mucosa, while pro-cholinergic drugs accelerate the gastrointestinal transit time and increase secretion [53].

Treatment of celiac disease: The standard treatment for celiac disease is a gluten-free diet [7]. In our study, all patients with ICI-CD responded well to a gluten-free diet. However, just a symptomatic response with a gluten-free diet is not sufficient to make a diagnosis of celiac disease [19]. Additionally, on follow-up, a negative celiac serology does not guarantee mucosal healing. A repeat biopsy showing the return of the normal histology of duodenal mucosa is important to confirming disease resolution [7]. The long-term sequelae of untreated celiac disease can lead to adenocarcinoma of the small intestine, enteropathy-associated T-cell lymphoma and refractory sprue. Therefore, it may be crucial to adhere to a gluten-free diet once diagnosed with celiac disease [14].

Treatment of ICI-CD: A gluten-free diet may be sufficient to manage ICI-CD without the need for immunosuppression. On the other hand, systemic immunosuppression is required to treat ICI-EC [14]. Theodoraki et al. reported a case of a patient with ICI-EC with negative celiac serology, who improved only with gluten withdrawal. This is the only documented case where a gluten-free diet led to an improvement of ICI-EC [21]. In our study, we observed that many patients with ICI-CD were treated with systemic glucocorticoids first. However, these patients failed to improve with steroids alone. After stopping the ICI therapy, a gluten-free diet was adequate for symptomatic improvement in all patients. Some patients improved with only a gluten-free diet, whereas others were given concurrent therapy with steroids. Further studies are required to determine if it is solely a gluten-free diet that leads to clinical improvement or a combination of discontinuing ICI therapy, gluten-free diet and steroid therapy.

Reinitiating ICI therapy after clinical improvement: Symptomatic improvement occurred between 1 and 21 days from symptom onset in the ICI-CD cohort in our study. The choice of reinitiating treatment with ICIs in patients with ICI-induced toxicities depends on multiple factors, including the clinical response of the malignancy to the initial immunotherapy regimen, the severity of the ICI-induced toxicity, its response to treatment and the availability of alternative treatment options for the primary malignancy [54]. In our study, two patients with ICI-CD restarted ICI therapy after clinical improvement. However, despite adhering to a gluten-free diet, one of the patient’s symptoms relapsed. Further studies are required to determine the safety of resuming ICI therapy in patients with ICI-CD.

## 5. Limitations of the Study

This study is retrospective and therefore may be subject to biases. Additionally, the sample size was small due to the rarity of this condition. The findings from this study require prospective verification with a larger sample size.

## 6. Conclusions

Celiac disease is a rare gastrointestinal adverse effect of immune checkpoint inhibitor therapy. ICIs lead to immune dysregulation and thereby may unmask an inherited, underlying, asymptomatic celiac disease. This may be confirmed by performing celiac serology or an upper gastrointestinal endoscopy to diagnose celiac disease before initiating ICI therapy. Patients with pre-existing autoimmune diseases (especially type-1 diabetes mellitus) or a known HLA-DQ2-positive status may be more prone to suffer from ICI-induced celiac disease. Therefore, testing for HLA-DQ2 should be considered if ICI-associated gastrointestinal toxicity is suspected. Patients often present with non-bloody diarrhea. This condition is highly under-recognized and often misdiagnosed as ICI-induced enterocolitis. As a result, most patients may be initially treated with systemic steroids, which is the standard management of ICI-induced enterocolitis. However, we observed that in patients with ICI-induced celiac disease, discontinuing ICI therapy and introducing a gluten-free diet leads to rapid symptomatic improvement, and immunosuppressive therapy like steroids may not be as useful. There are other clinical findings that can help distinguish ICI-induced celiac disease from the more common ICI-induced enterocolitis, including the absence of abdominal pain, the presence of duodenal villous blunting and colonic cryptitis. The gold standard for diagnosing ICI-induced celiac disease is a positive celiac serology. Based on the results of our study, we recommend that patients receiving ICI therapy presenting with non-bloody diarrhea be tested for celiac disease, especially if there is no improvement with steroids and other immunosuppressive medication. If celiac disease is confirmed, a gluten-free diet should be implemented. A gluten-free diet is a cost-effective and efficient treatment modality which spares the patient from unnecessary exposure to the adverse effects of systemic immunosuppression while leading to the rapid improvement of symptoms. The early recognition of this disease can help avoid future complications and the discontinuation of antineoplastic immunotherapy.

## Figures and Tables

**Table 1:** Demographic, Clinical and Pathological features of patients with ICI-induced celiac disease and ICI-induced enterocolitis.

	ICI-Induced Celiac Disease (Cases)	ICI-Induced Enterocolitis (Controls)
No. of Subjects	8	24
Age	62–79 years	22–83 years
Median age	70 years	65 years
<65 years (%)	2 (25)	12 (50)
>65 years (%)	6 (75)	12 (50)
Sex		
Male (%)	5 (62.5)	18 (75)
Female (%)	3 (37.5)	6 (25)
Race/Ethnicity		
Caucasian (%)	4 (50)	5 (20.83)
Asian (%)	0	1 (4.17)
Not specified	4 (50)	18 (75)
Preexisting Autoimmune Disease		
Yes (%)	2 (25)	1 (4.17)
Thyroid	1 (12.5)	0
Diabetes Mellitus (Type 1)	1 (12.5)	1 (4.17)
No (%)	6 (75)	23 (95.83)
HLA-DQ2 Testing		
Positive	3 (37.5)	0
Negative	0	0
Not performed	5 (62.5)	24 (100)
Primary Malignancy		
Genitourinary (%)	2 (25)	4 (16.67)
Clear Cell Renal Cell Carcinoma (%)	1 (12.5)	4 (16.67)
Prostate Adenocarcinoma (%)	1 (12.5)	0
Lung (%)	1 (12.5)	5 (20.83)
Non-small cell (%)	1 (12.5)	2 (8.33)
Adenocarcinoma	0	3 (12.5)
Malignant Melanoma (%)	3 (37.5)	12 (50)
Gastrointestinal (%)	0	2 (8.33)
Colon (%)	0	1 (4.17)
Esophageal (%)	0	1 (4.17)
Other (%)	2 (25)	1 (4.17)
Lobular Breast Adenocarcinoma (%)	1 (12.5)	0
Mesothelioma (%)	1 (12.5)	0
Head and Neck Squamous Cell Carcinoma (%)	0	1 (4.17)
Stage of primary malignancy at the time of starting ICI therapy		
1 (%)	1 (12.5)	1 (4.17)
2 (%)	1 (12.5)	0
3 (%)	1 (12.5)	4 (16.67)
4: Distant Metastasis (%)	4 (50)	18 (75)
Not specified (%)	1 (12.5)	1 (4.17)
Treatment of malignancy before initiating ICI therapy		
Resection (%)	3 (37.5)	9 (37.5)
Chemotherapy (%)	3 (37.5)	7 (29.17)
Radiation (%)	3 (37.5)	4 (16.67)
Immunotherapy (%)	1 (12.5)	5 (20.83)
Non-ICI agent (%)	0	1 (4.17)
ICI (%)	0	3 (12.5)
Combination of both (%)	1 (12.5)	1 (4.17)
ICI used		
Ipilimumab (%)	2 (25)	6 (25)
Pembrolizumab (%)	2 (25)	7 (29.17)
Combination of Ipilimumab and Nivolumab (%)	2 (25)	7 (29.17)
Nivolumab (%)	1 (12.5)	4 (16.67)
Durvalumab (%)	1 (12.5)	0 (0)
Type of ICI used		
CTLA-4 Inhibitor (%)	2 (25)	6 (25)
PD-1/PDL-1 Inhibitor (%)	4 (50)	11 (45.83)
Combination of CTLA-4 and PD-1 Inhibitors (%)	2 (25)	7 (29.17)
No. of cycles of ICI therapy prior to onset of diarrhea	1–5	1–13
1 (%)	2 (25)	6 (25)
2 (%)	4 (50)	5 (20.83)
3 (%)	0	6 (25)
4 (%)	1 (12.5)	2 (8.33)
≥5 (%)	1 (12.5)	2 (8.33)
Not specified (%)	0	3 (12.5)
>2 cycles (%)	2 (25)	10 (41.66)
Median time between first dose of ICI and onset of diarrhea (weeks)	3 (1–15)	6 (1–40)
ICI therapy continued despite onset of diarrhea (%)	4 (50)	3 (12.5)
Grade of Diarrhea		
1 (%)	2 (25)	0
2 (%)	0	4 (16.67)
3 (%)	3 (37.5)	11 (45.83)
4 (%)	3 (37.5)	5 (20.83)
Not specified (%)	0	4 (16.67)
Other Clinical Manifestations		
Abdominal Pain	1 (12.5)	15 (62.5)
Nausea/Vomiting (%)	2 (25)	4 (16.67)
Weight Loss (%)	5 (62.5)	5 (20.83)
Nutritional/Electrolyte Deficiency (%)	6 (75)	7 (29.17)
Other ICI-related adverse effect		
Yes (%)	2 (25)	9 (37.5)
Thyroiditis (%)	1 (12.5)	3 (12.5)
Transaminitis (%)	1 (12.5)	2 (8.33)
Pancreatitis (%)	1 (12.5)	1 (4.17)
Involvement of skin (%)	0	1 (4.17)
Adrenal Insufficiency (%)	0	1 (4.17)
Interstitial Pneumonitis (%)	0	1 (4.17)
No (%)	6 (75)	15 (62.5)
Duodenal Microscopy		
Villous Atrophy (%)	8 (100)	4 (16.67)
Crypt Hyperplasia (%)	3 (37.5)	2 (8.33)
Expansion of Lamina Propria (%)	2 (25)	1 (4.17)
Immunostaining positive for CD3 (%)	2 (25)	1 (4.17)
Endoscopy not performed (%)	0	18 (75)
Colonic Microscopy		
Crypt Abscess/Cryptitis (%)	0	13 (54.16)
Lamina Propria Inflammatory Infiltrates (%)	0	13 (54.16)
Epithelial apoptotic bodies (%)	1 (12.5)	5 (20.83)
Colonoscopy not performed (%)	2 (25)	6 (25)

**Table 2:** Treatment of patients with ICI-induced celiac disease compared to ICI-induced enterocolitis.

Treatment	ICI-Induced Celiac Disease (Cases)	ICI-Induced Enterocolitis (Controls)	*p* Value of Chi Square
No. of subjects	8	23	
First-Line Treatment			0.007 0.116
Systemic Glucocorticoids (%)	5 (62.5)	21 (91.3)	
Budesonide (%)	0	1 (4.34)	
Gluten-Free Diet (%)	3 (37.5)	0	
Loperamide (%)	0	1 (4.34)	
5-Aminosalicylate (%)	0	1 (4.34)	
Failure of First-Line Treatment			
Systemic Glucocorticoids (%)	3 (60)	9 (42.85)	0.261
Gluten-Free Diet (%)	0	N/A	N/A
Others (%)	N/A	1 (50)	N/A
Second-Line Treatment			
Systemic Steroids (%)	2 (12.5)	4 (17.39)	
Budesonide (%)	1 (12.5)	1 (4.34)	
Gluten-Free Diet (%)	5 (62.5)	0	
Infliximab (%)	0	6 (26.09)	
Others (%)	0	1 (4.34)	
Mesalamine (%)	0	1 (4.34)	
Octreotide (%)	0	1 (4.34)	
Not required (%)	1 (12.5)	13 (56.52)	
Failure of Second-Line Treatment			
Systemic Steroids (%)	0	1 (25)	
Gluten-Free Diet (%)	0	N/A	
Infliximab (%)	N/A	1 (16.67)	
Others (%)	N/A	1 (100)	
Dose of Systemic Steroids given (mg/day in prednisone units)	50 (3.8–150)	65 (30–160)	
Biologicals			
Required (%)	0	9 (39.13)	
Infliximab (%)	0	8 (34.78)	
Vedolizumab (%)	0	3 (13.04)	
Tacrolimus (%)	0	1 (4.34)	
Did not require (%)	8 (100)	14 (60.87)	
Median time required for symptomatic improvement after starting treatment (days)	14 (0–21)	4 (1–42)	
Restarted ICI after improvement of diarrhea (%)	2 (25)	3 (13.04)	
Relapse of diarrhea with reinitiating ICI despite adhering to treatment (%) *	1 (50)	0	N/A
